# The effect of path length, light intensity and co-added time on the detection limit associated with NIR spectroscopy of potassium hydrogen phthalate in aqueous solution

**DOI:** 10.1371/journal.pone.0176920

**Published:** 2017-05-04

**Authors:** Tetsuya Inagaki, Tomoko Watanabe, Satoru Tsuchikawa

**Affiliations:** Graduate School of Bioagricultural Sciences, Nagoya University, Furo-cho, Chikusa-ku, Nagoya, Japan; Aligarh Muslim University, INDIA

## Abstract

Near infrared (NIR) spectroscopy is a common means of non-invasively determining the concentrations of organic compounds in relatively transparent aqueous solutions. Rigorous determination for limit of detection (LOD) is of importance for the application use of NIR spectroscopy. The work reported herein determined the LOD with the analysis of potassium hydrogen phthalate (KHP) in water with partial least square (PLS) calibration in the range of 6300–5800 cm^-1^ between the two strong absorption bands of water, in which the C-H overtone bands of KHP are located. A comparison of the LOD estimated when using various condition (path length, aperture and co-added scan times) showed that the lowest LOD for KHP obtained with a fiber optic cable attachment equipped NIR spectrometer is approximately 150 ppm.

## Introduction

Near-infrared (NIR) spectroscopic techniques can be utilized for the non-invasive quantification of multiple analytes in aqueous materials [1–4]. Due to the low absorptivity of water molecules in the NIR region compared to IR region, NIR spectroscopy allows longer optical path lengths to be used when studying aqueous solutions as compared to other techniques, and thus is useful when studying water-solute interactions [5, 6]. However, the absorption intensity of an analyte in solution will generally be weak compared to the intensity of the broad absorption bands of the aqueous solvent, because the volume ratio of analyte in aqueous solvent is relatively very small compared to that of aqueous solvent. Furthermore, the water absorption bands are significantly affected by variations in temperature. Regardless, many studies have emphasized the feasibility of analyzing various analytes in water at low concentrations, down to the ppm level. Ding et al. showed the possibility of determining the concentrations of methyl iso-butyl ketone (MIBK) and tri-butyl phosphate (TBP) in aqueous solutions over the range of 1–100 ppm by NIR spectroscopy [7] while Mauer et al. reported the detection of 1 ppm melamine in milk powder using NIR [8]. These studies employed the root mean square error of cross validation (*RMSECV*) or root mean square error of validation (*RMSEV*) calculated from partial least square (PLS) or other multivariate regression techniques based on factor analysis, with no restrictions on the number of wavelengths that could be selected for the calibration. These parameters allow the calibration algorithm to extract the maximum information from the spectra. Multivariate regression analysis is generally suitable for the evaluation of complex spectral signals such as those present in NIR data. For the PLS regression analysis, as factors which are unrelated to the independent variable (such as fluctuations in the light intensity output of the spectrometer) may be incorporated into the calibration set, it is very important to decide optimum number of latent variable rejecting the miss-contribution of unrelated factors. Generally, leave-one-out cross validation are used for determination of optimum number. Haaland et al. suggested to use *F* statistic for selecting optimum number showing such that minimum prediction error sum of squares (PRESS) for that model is not significantly greater than the minimum PRESS [9].

The limit of detection (LOD) of an analyte should correspond to a concentration which produces a spectral signal for that analyte which significantly differs from the signal obtained from a blank sample, or from the background signal. LOD of an analyte is therefore governed by the signal to noise (S/N) ratio. Although there are many parameters affecting the S/N ratio, the key factors are the wavenumber region, optical path length, co-adding scan times and light intensity from light source. In a given wavenumber region, the absorption intensity of water will determine the optical path length of the cell which will allow precise measurements. It is in fact well-known that the optical path length strongly influences the S/N ratio and some researchers have examined the relationship between optical path length and S/N ratio in great detail [10–13]. Jensen et al. determined the noise levels associated with eight different optical path lengths, ranging from 0.2 to 2.0 mm, using both pure water and a 1 g/dL aqueous glucose solution and concluded that the noise levels in the spectral region from 5000 to 4000 cm^-1^ indicated that the optimal optical path length of 0.4 mm was the same for pure water and aqueous glucose solutions [12].

For the determination of LOD, concept of types I and II errors (false positives and false negatives), on the propagation of uncertainties in slope and intercept should be taken into account as the International Union of Pure and Applied Chemistry (IUPAC) recommended [14–16]. Allegrini et al. suggested IUPAC-consistence approach for LOD proposed for PLS multivariate calibration [17]. We applied the method suggested by them for evaluation of LOD. Several concentrations of an aqueous potassium hydrogen phthalate (KHP) solution were used for the trials. KHP was used in this study as an anlyte chemical because we are trying to introduce NIR spectroscopy for the monitoring of pollution degree in sewage treatment plants as many researchers showed the high potential of NIR spectroscopy for that purpose [8]. KHP is a standard employed in sewage treatment plants to determine total organic carbon (TOC) which is one of the important pollution degree used in sewage treatment plant in Japan [18]. LOD was evaluated using transmittance spectra of these aqueous solutions with aid of PLS regression analysis. Although, LOD of analyte highly depend of the spectrometer performance (i.e. spectroscopic system like Fourier transform (FT) or dispersive grating, existence of optical fiber), we tried to rigorously determine LOD using FT-spectrometer equipped with optical fiber.

## Material and method

### Sample preparation

KHP (204.2236 g mol^-1^) was obtained from standard chemical supply sources and used without further purification. KHP is a standard chemical employed in sewage treatment plants to determine total organic carbon (TOC) which is one of the important pollution degree used in sewage treatment plant in Japan. A total of 38 samples of KHP in purified water were prepared over the range of 1 to 10,000 ppm in logarithmically increasing concentration increments (0, 1, 2, 3, 4, 5, 6, 7, 8, 9, 10, 20, 30, 40, 50, 60, 70, 80, 90, 100, 200, 300, 400, 500, 600, 700, 800, 900, 1000, 2000, 3000, 4000, 5000, 6000, 7000, 8000, 9000 and 10000 ppm). These concentrations were calculated on a weight/volume basis for the amount of carbon in each solution, meaning that one liter of the 1 ppm solution contained 0.001 g of carbon (0.002127 g of KHP). Each solution was prepared by diluting a 10000 ppm stock solution with purified water. We calculated the adjustment error in the concentration of each sample using error propagation equation from the error for laboratory glasswares. The adjustment error for all samples were smaller than 1.5% of concentrations.

### Spectral measurement

In this study, aqueous solution transmission spectra were obtained using FT-NIR spectrometer (Matrix-F: Bruker, Massachusetts) having a fiber optic cable attachment (P600-025-VIS-NIR: Ocean optics, Florida) equipped with a water jacketed cuvette holder (CUV-UV: Ocean optics, Florida) that allowed temperature control to within ±0.1°C. The empty cell was used for reference measurements. The sample temperature in the quartz cell was controlled to 30°C. The spectrometer was equipped with a rectangular quartz beam splitter and a TE-InGaAs detector. Spectra were acquired over the range of 8,000 to 5000 cm^-1^ using optical path lengths of 1, 2, 5 and 10 mm. The wavenumber resolution was set to 8 cm^-1^. Double sided forward backward interferograms were collected at a 10 kHz scan velocity and Blackman-Harris apodization and Mertz phase correction were applied. During Fourier processing to produce spectra, 2 level zero-filling was used (corresponding to the wavenumber interval of 4 cm^-1^). Transmission measurements of the aqueous KHP solutions were performed with the samples held in rectangular quartz cells with various optical path lengths. Spectra were co-added for 8, 16, 32, 64 and 128 times, respectively, and averaged. We employed three kinds of aperture (BRM2065, NG9, NG11) to change the light intensity from light sources. These aperture are equipped in the spectrometer for the wavenumber proof (BRM2065 is glass filter containing rare-earth oxide, which have the absorption bands at specific wavenumbers) and absorbance proof (NG9 and NG 11 are the glass filter), respectively. Although these aperture are not used for common measurement, we employed these to get different kind of light intensity. Briefly, we measured each samples under 60 measurement conditions (4 pathlengths × 3 apertures × 5 co-added scan times). We tested samples randomly with respect to concentration and three replicate spectra were collected for each sample. OPUS software (Bruker, Germany) was used for spectral measurement and MATLAB (MathWorks, Massachusetts) was used for spectral analysis.

## Result and discussion

### Absorbance spectra of the analyte

**Fig 1:**
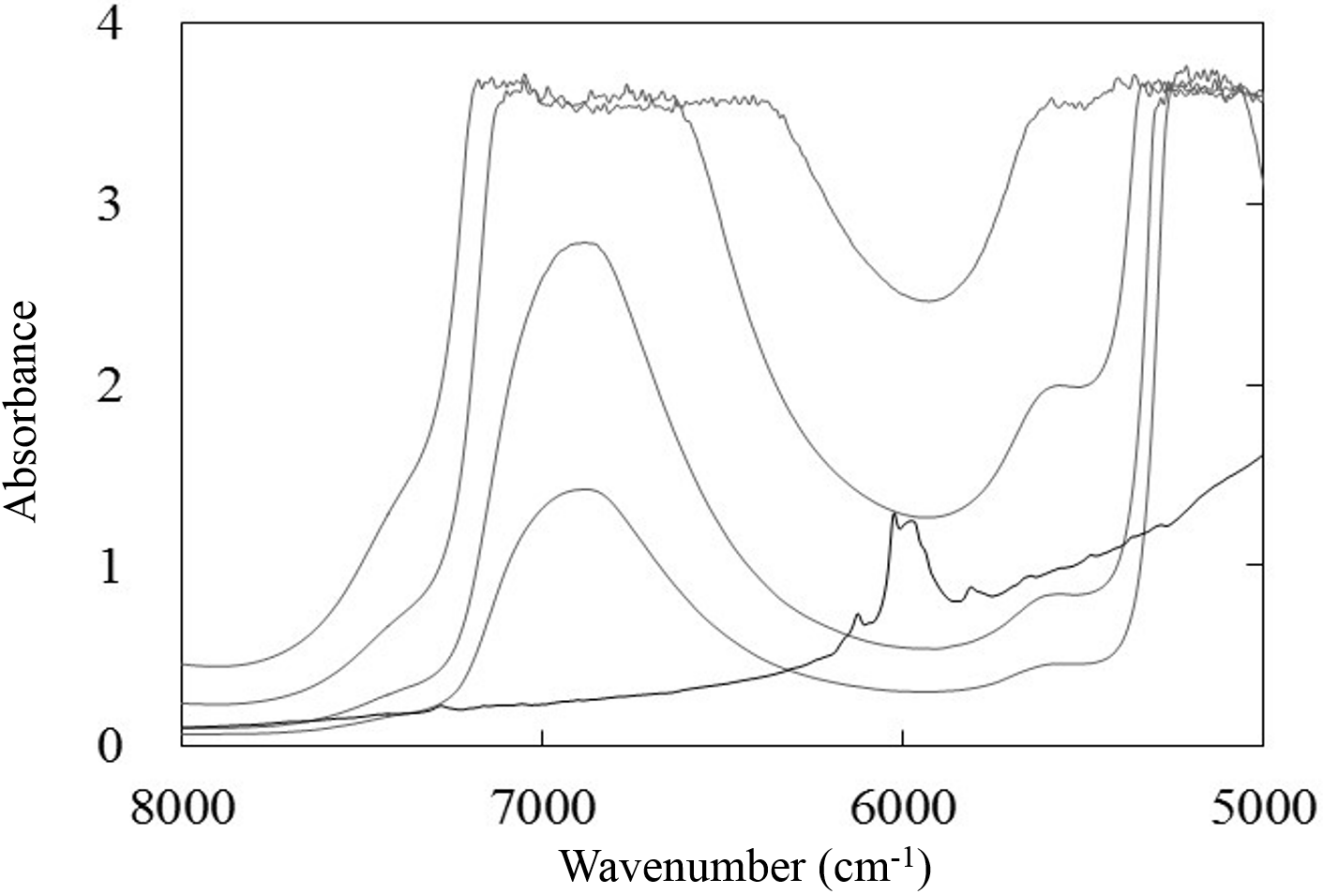
NIR spectra of purified water (gray lines) at various path lengths (1, 2, 5, 10 mm) and powdered KHP (black line).

The quantitative determination of KHP in water requires the identification of spectral bands related to the analyte, even though these bands will be embedded in the strong absorbance spectrum of the solvent. Fig 1 shows the NIR spectra of distilled water (gray solid lines) measured in the transmittance mode at several optical path lengths, as well as that of KHP powder (black solid line) measured in the diffuse reflectance mode. It is evident that there is a spectral window, ranging from 6300 to 5800 cm^-1^, between the two strong absorption bands of water, in which the C-H overtone bands of KHP are located. This spectral region is therefore useful for the quantitative analysis of KHP.

### Determination of the LOD_max_ for KHP

**Fig 2:**
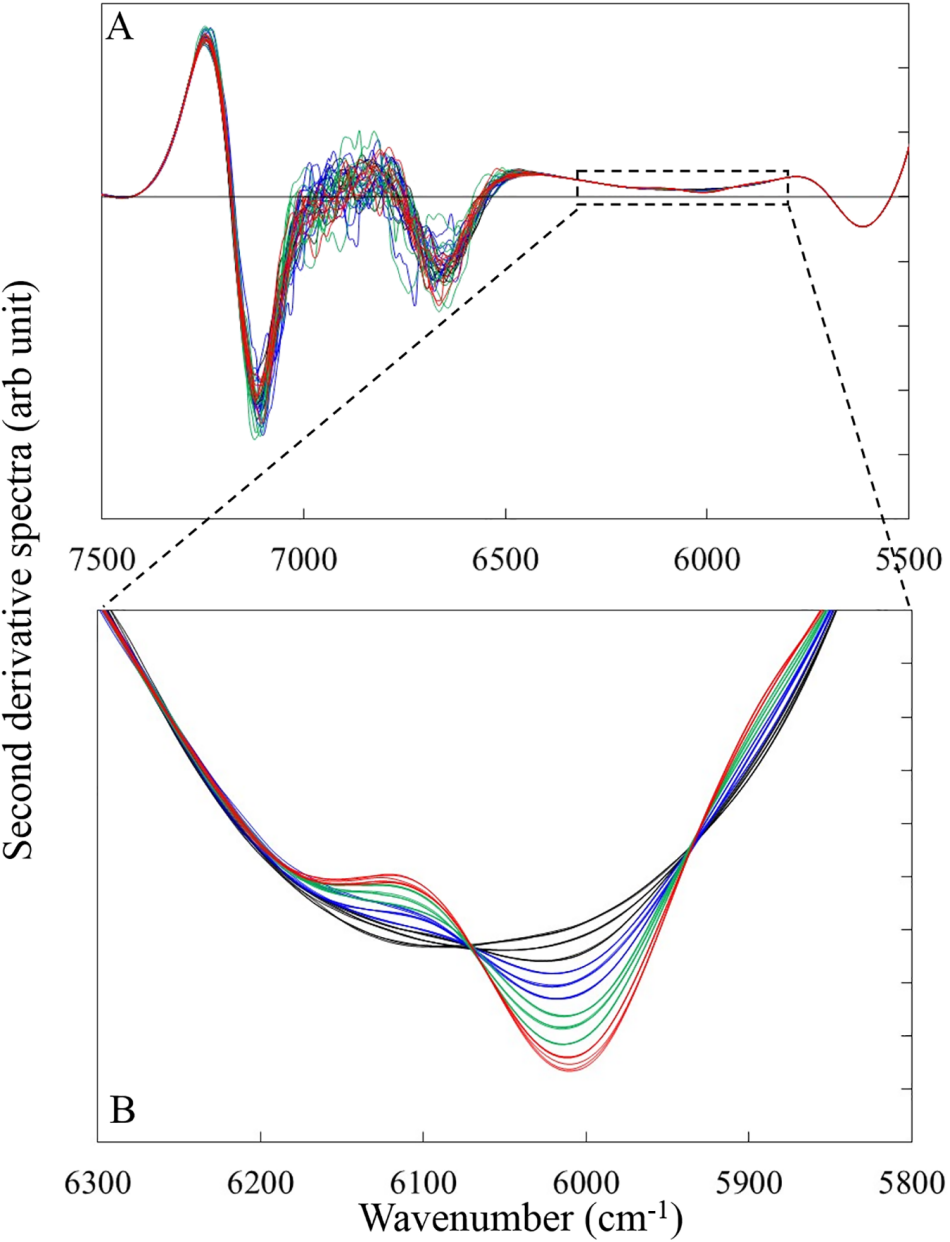
NIR second derivative spectra (gap-segment, 23 segments) KHP aqueous solutions at the wavenumber range of (A) 7500–5500 cm^-1^ and (B) 6300–5800 cm^-1^. Black line is the spectra of 0, 1000 and 2000 ppm solution, blue line is that of 3000, 4000 and 5000 ppm, green line is that of 6000, 7000, 8000 ppm and red line is that of 9000 and 10000 ppm.

We examined the effects of optical path lengths, aperture type and co-added scan time on the LOD of KHP. Fig 2 shows the NIR second derivative spectra (gap-segment, 23 segments) of KHP aqueous solutions. The amplitude of the second derivative values at around 6000 cm^-1^ increased with the increase of KHP concentration (Fig 2B). This band is assigned to the first overtone of the CH group in KHP.

Several calibration equations between absorbance at the wavelength range of 6300–5800 cm^-1^ and KHP concentration were determined using PLS with different spectral pre-treatment. The spectral pre-treatments employed in this study were: (1) no pre-treatment, (2) linear baseline correction, (3) multiplicative scattering correction (MSC), (4) second derivative (Savitzky-Golay: 3–101 smoothing points) and (5) second derivative (gap-segment: 3–101 segments). As the calibration equation yielding the highest determination coefficient (*r*^2^) was obtained when applying spectral pre-treatment consisting of the second derivative with a gap segment (23 smoothing points), which was independent of the optical path length, co-added scan time and aperture, we used gap segment second derivative (23 smoothing points) for the calculation of LOD. Leave-one-out cross validation were used for determination of optimum number as Haaland et al. suggested using *F* statistic showing such that PRESS for that model is not significantly greater than the minimum PRESS [9]. We used *F* value for 95% percentile of Snedecor’s *F* distribution with the number of sample degrees of freedom. Determination coefficient (*r*^2^) and PRESS were calculated as follows;

$$r^{2}=1-\frac{\sum_{i}^{}{\left(y_{i}-\tilde{y_{i}}\right)}^{2}}{\sum_{i}^{}{\left(y_{i}-\bar{y}\right)}^{2}}$$ (1)

$$PRESS=\frac{1}{I}\sum_{i}^{}{\left(y_{i}-{\tilde{y}}_{i}\right)}^{2}$$ (2)

where *y*_i_ is the concentration of KHP in sample *i*, $\bar{y}$ is the average concentration, ${\tilde{y}}_{i}$ is the KHP concentration predicted by the PLS regression line for sample *i*, *I* is the number of calibration samples. LOD was estimated by the method according to Allegrini et al. [17]. They proposed the equation calculating the lower and upper limit of the LOD interval (LOD_min_ and LOD_max_) correspond to the PLS calibration samples with the lowest and largest extrapolated leverages to zero analyte concentration. It can be regarded that analyte is not detected in a given sample if its predicted value is below LOD_min_, or that it is present if its predicted concentration is above LOD_max_. LOD_min_ and LOD_max_ was estimated by

$$LOD_{\min}=3.3{\left[SEN^{-2}\mathrm{var}(x)+h_{0\min}SEN^{-2}\mathrm{var}(x)+h_{0\min}\mathrm{var}(y_{cal})\right]}^{1/2}$$ (3)

$$LOD_{\max}=3.3{\left[SEN^{-2}\mathrm{var}(x)+h_{0\max}SEN^{-2}\mathrm{var}(x)+h_{0\max}\mathrm{var}(y_{cal})\right]}^{1/2}$$ (4)

where SEN is the sensitivity given in PLS by the inverse of the length of the regression coefficients, var(*x*) is the variance in instrumental signals, var(*y*_cal_) the variance in the calibration concentrations. *h*_0min_ and *h*_0max_ are minimum and maximum value for sample leverage suggested by Allegrini et al. [17]. Because mean centering for spectral data were employed in this study, effective leverage (*h*_0min_+1/*I*) and (*h*_0max_+1/*I*) are used. The variance in spectral signals var(*x*) was estimated from the sequential measurement of pure water (KHP concentration is 0 ppm) 10 for each of 60 measurement conditions (4 pathlengths × 3 apertures × 5 co-added scan times). Variance in concentrations var(*y*) were estimated by uncertainty propagation analysis from the uncertainties in the chemistry apparatus used for the sample preparation starting from anlyte standards.

**Fig 3:**
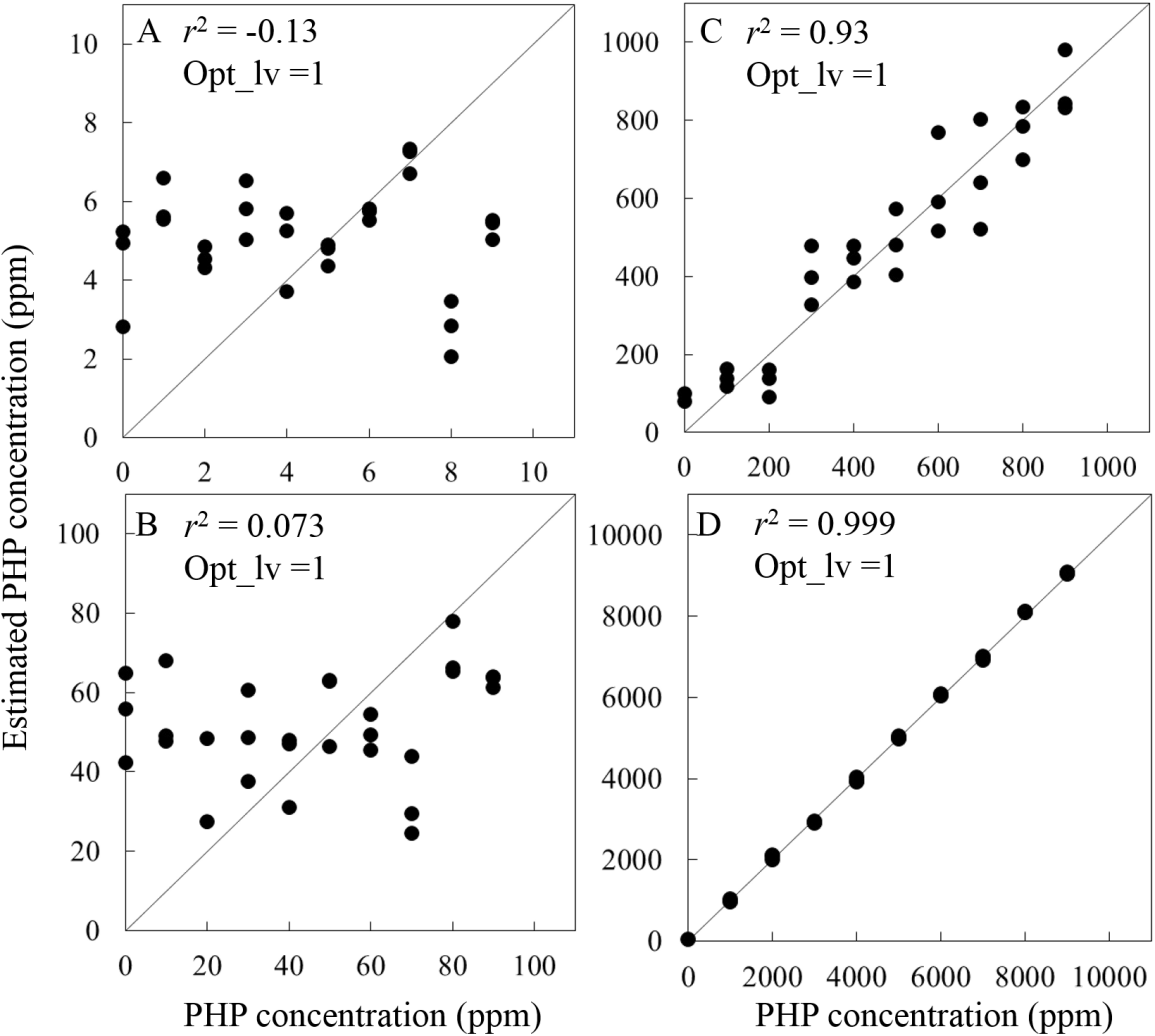
Relationships between measured and predicted KHP concentration (spectral pre-treatment: gap-segment second derivative, 23 segments, optical path length: 5 mm, aperture: BRM2065, co-added time: 32 scans) at incorporating the data for (A) 0–10 ppm (1 ppm steps), (B) 0–100 ppm (10 ppm steps), (C) 0–1000 ppm (100 ppm steps), (D) 0–10000 ppm (1000 ppm steps).

PLS regression analysis and LOD estimation were done using spectra obtained with KHP concentrations in the ranges of 0–10 ppm (in 1 ppm steps), 0–100 ppm (in 10 ppm steps) 0–1000 ppm (in 100 ppm steps) and 0–10000 ppm (in 1000 ppm steps). Regression coefficient calculated by PLS regression using the samples of which the concentration range is 0–10000 ppm (in 1000 ppm steps) was used for the calculation of LOD for all measurement condition because of the highest *r*^2^ value. The best LOD_max_ = 156.2 ppm, in the 60 kinds of spectral measurement condition, was acquired when spectra measured at the 5 mm pathlength using BRM2065 aperture with 32 co-added scan times (second derivative signal (gap-segment 23 segments)) when the samples KHP concentration range of 0–10000 ppm (in 1000 ppm steps). Fig 3 shows the relationship between the measured and predicted concentration of KHP and the second derivative (spectral pre-treatment: gap-segment second derivative, 23 segments, optical path length: 5 mm, aperture: BRM2065, co-added time: 32 scans). Suggested optimum number of latent variable by *F*-test was 1 for all KHP ranges. As expected, *r*^2^ increased as the concentration range was increased. There is a significant correlation between measured and predicted concentration in the range of 0–1000 ppm (Fig 3C) with *r*^2^ of 0.93. LOD_max_ calculated when using spectra obtained with KHP concentrations in the ranges of 0–10000 ppm (Fig 3D) was 156.2 ppm. The value is well corresponding to the *r*^2^ values calculated using each KHP concentration range (i.e. *r*^2^ values were low at the concentration smaller than 100 ppm (Fig 3A and 3B), although *r*^2^ values were very high at the concentration bigger than 100 ppm (Fig 3C and 3D).

Many research predicting total carbon content in soil by NIR reflectance spectroscopy have been reported [19, 20]. Chang et al. reported the *RMSECV* value of 7.86 g kg^-1^ with 0.87 *r*^2^ for the prediction of total carbon content in soil ranging between 1.3–285.8 g kg^-1^ by NIR reflection spectroscopic measurement with aid of PLS regression analysis [19]. The value acquired in this study (LOD_max_ = 156.2 ppm), which is much better than their study, imply that effect noise or unrelated factor on the reflectance spectra in soil sample is significant compared to that in transmission measurement of aqueous solution. For the aqueous solution measurement, Ding et al. reported the SEP value of 3.82 ppm for the MIBK ranging between 1–160 ppm using transmission spectra between 5000–4000 cm^-1^ measured by FT-NIR spectrometer with enough *r*^2^ value [7]. The LOD_max_ calculated from the spectra obtained in this study was higher than resulting from such a result due to the external fiber optic cable attached. As the value of LOD_max_ = 156.2 ppm is not enough for the quality control in the swage factory, we should use the NIR spectrometer equipping the sample compartment without optical fiber for this purpose having better S/N ratio.

### Change of LOD_max_ as a function of optical path length

Since the LOD_max_ is determined directly by the S/N ratio and indirectly by the molar absorptivity of the analyte, the optical path length is an important factor which impacts both S/N ratio and signal intensity. In Table 1, statistical results obtained from LOD_max_ at several optical path lengths (spectral pre-treatment: gap segment second derivative, data set: all samples, aperture: BRM2065, co-added scan time: 32 scans) are shown in 2–5 lines from the top.

**Table 1:** Statistical results of PLS regression analysis and LOD_max_.

Path length (mm)	Aperture	Co-added	*r*^*2*^	SEN	var(*x*)	LOD_max_
1	BRM2065	32	0.99	2.7E-06	4.6E-07	881.4
2	BRM2065	32	0.995	5.1E-06	1.0E-07	224.2
5	BRM2065	32	0.999	1.3E-05	3.3E-07	156.2
10	BRM2065	32	0.97	2.5E-05	7.0E-05	1154.7
5	BRM2065	32	0.999	1.3E-05	3.3E-07	156.2
5	NG9	32	0.998	1.3E-05	1.1E-06	283.6
5	NG11	32	0.9994	1.3E-05	4.4E-07	177.8
5	BRM2065	8	0.999	1.3E-05	5.6E-07	200.1
5	BRM2065	16	0.9996	1.2E-05	4.0E-07	187.6
5	BRM2065	32	0.999	1.3E-05	3.3E-07	156.2
5	BRM2065	64	0.9992	1.3E-05	5.7E-07	205.1
5	BRM2065	128	0.9997	1.3E-05	2.0E-06	379.2

*r*^2^:determination coefficient, SEN: sensitivity given in PLS by the inverse of the length of the regression coefficients, var(*x*): variance in instrumental signals.

**Fig 4:**
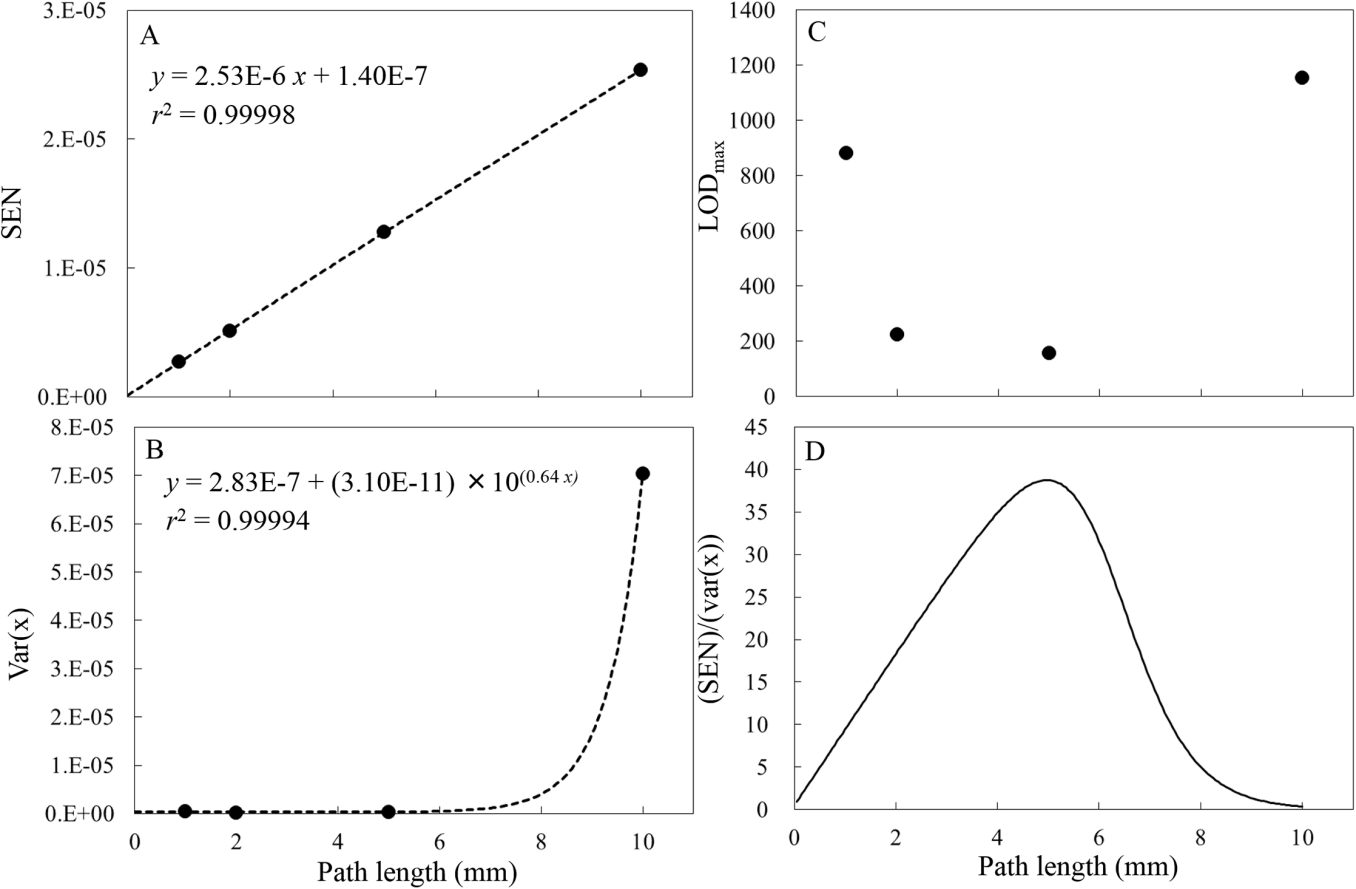
Variations in (A) SEN, (B) var(*x*), (C) LOD_max_ and (D) the regression line for SEN divided by the var(*x*) regression line as a function of optical path length (spectral pre-treatment: gap-segment second derivative, 23 segments, aperture: BRM2065, co-added time: 32 scans).

Fig 4 summarizes the variation with path length of (A) SEN (B) var(*x*) (C) LOD_max_ value calculated by Eq (4). The path length of 5 mm yielded the optimal value of LOD_max_ (Fig 4C). The SEN value (Fig 4A) is observed to increase in a linear fashion with optical path length, in accordance with the Lambert-Beer law (although the SEN is the inverse of the length of the regression coefficients in the wavenumber range of 6300–5800 cm^-1^), generating regression lines with the equations SEN = 2.53E-6×*L* + 1.40E-7 (where *L* is the optical pathlength), with associated determination coefficients of 0.99998. The absorbance (A) at a path length of *L* can be expressed by the Lambert-Beer Law.

$$\text{A}=-{\text{log}}_{10}\left(\frac{I}{I_{0}}\right)=\varepsilon \text{cL}$$ (5)

Here, *ε* is the molar absorptivity, *c* is the molar concentration of the analyte and *I* and *I*_*0*_ are the measured sample and reference intensities, respectively. The values of var(*x*) corresponding to the standard variance of the blank (or spectral noise) are seen to increase exponentially with increasing optical path length, as a result of the increasing intensities of the two strong water absorption bands at both sides of the spectral window being examined (6300–5800 cm^-1^). Based on error propagation, the noise or absorbance error may be expressed by:

$$\sigma_{\text{A}}{}^{2}=\sigma_{\text{I}}{}^{2}{\left(\frac{\partial \text{A}}{\partial \text{I}}\right)}^{2}+\sigma_{{\text{I}}_{0}}{}^{2}{\left(\frac{\partial \text{A}}{\partial {\text{I}}_{0}}\right)}^{2}$$ (6)

where ∂*I* and ∂*I*_0_ are the errors in *I* and *I*_0_, respectively. If we assume that *I*_0_ is much greater than *I*, ∂*I* and ∂*I*_0_ are equal to the detector noise (*n*_d_). We can obtain the following equation [11].

$$\sigma_{\text{A}}\approx \frac{\sqrt{2}}{\log10}\frac{{\text{n}}_{\text{d}}}{\text{I}}$$ (7)

Since Eq (5) suggests that *I* will decrease exponentially with optical path length, σ_A_^2^ should increase exponentially with optical pathlength, and this is confirmed in Fig 4B. The regression lines fitted to these data had the equations var(*x*) = 2.83E-7+3.10E-11×10^0.64L^ with *r*^2^ values of 0.99994. Optimal optical path lengths were determined by dividing the SEN regression line by the regression line of var(*x*) (see Fig 4D) and this calculation demonstrated that the optimal values were *L* = 4.95 mm. This calculated optical path lengths are almost the same as the experimentally determined value of *L* = 5 mm. This result suggests that, when ascertaining the optimal path length, the effects of SEN and spectral noise (var(*x*)) must be taken into consideration. Jensen et al. determined the noise levels associated with eight different optical path lengths, ranging from 0.2 to 2.0 mm, using both pure water and a 1 g/dL aqueous glucose solution and concluded that the noise levels in the spectral region from 5000 to 4000 cm^-1^ indicated that the optimal optical path length of 0.4 mm was the same for pure water and aqueous glucose solutions [12]. As SEN value, which is related to the molar absorption coefficient of analyte chemical, are taken into account for the calculation of LOD_max_ by Eq (4), it is possible to estimated specific optimal pathlength for KHP analyte.

### Change of LOD_max_ as a function of light intensity

**Fig 5:**
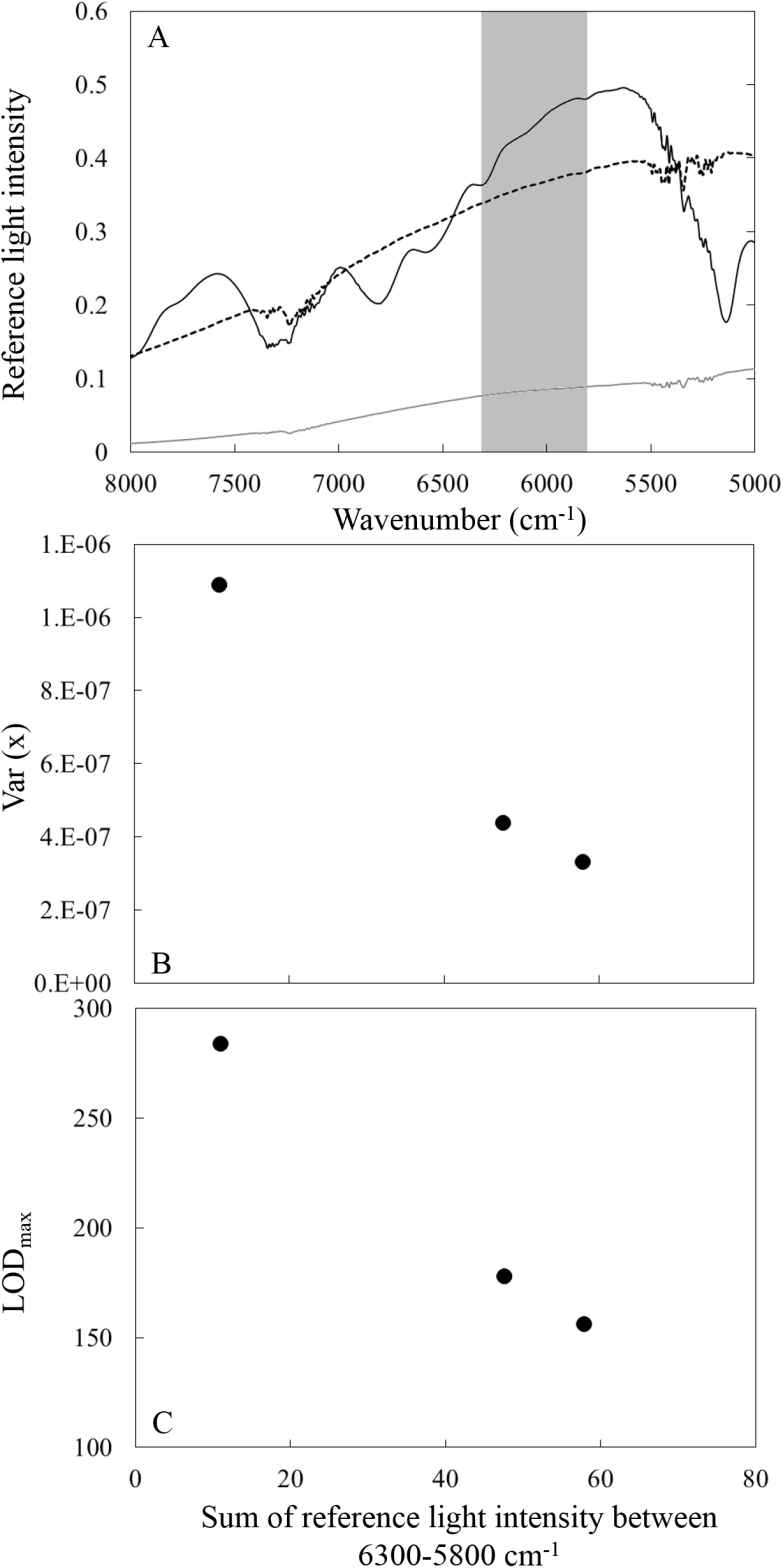
(A) Wavenumber dependent reference light intensity when aperture BRM2065 (black solid line), NG9 (gray solid line) and NG 11 (black dash line) were used. Variations in (B) var(*x*) and (C) LOD_min_ as a function of sum of reference sum of light intensity among 6300–5800 cm^-1^ (spectral pre-treatment: gap-segment second derivative, 23 segments, optical path length: 5 mm, co-added time: 32 scans).

We investigated the change of LOD_max_ as a function of reference light intensity, which directly effect on the S/N ratio. We employed the three kinds of apertures to change the reference light intensity. PLS calculations for three kinds of apertures (spectral pre-treatment: gap segment second derivative, pathlength: 5 mm, co-added scan time: 32 scans) are shown in 6–8 lines from the top in Table 1. Fig 5A shows the wavenumber dependent reference light intensity when aperture BRM2065 (black solid line), NG09 (gray solid line) and NG 11 (black dash line) were used. Fig 5 shows the change (B) var(*x*) and (C) LOD_max_ as a function of reference sum of light intensity between 6300–5800 cm^-1^ (graycolor masked in Fig 5A) used for the PLS regression analysis. Better var(*x*) and LOD_max_ were obtained when the reference light intensity was higher.

### Change of LOD_max_ as a function of co-added scan time

**Fig 6:**
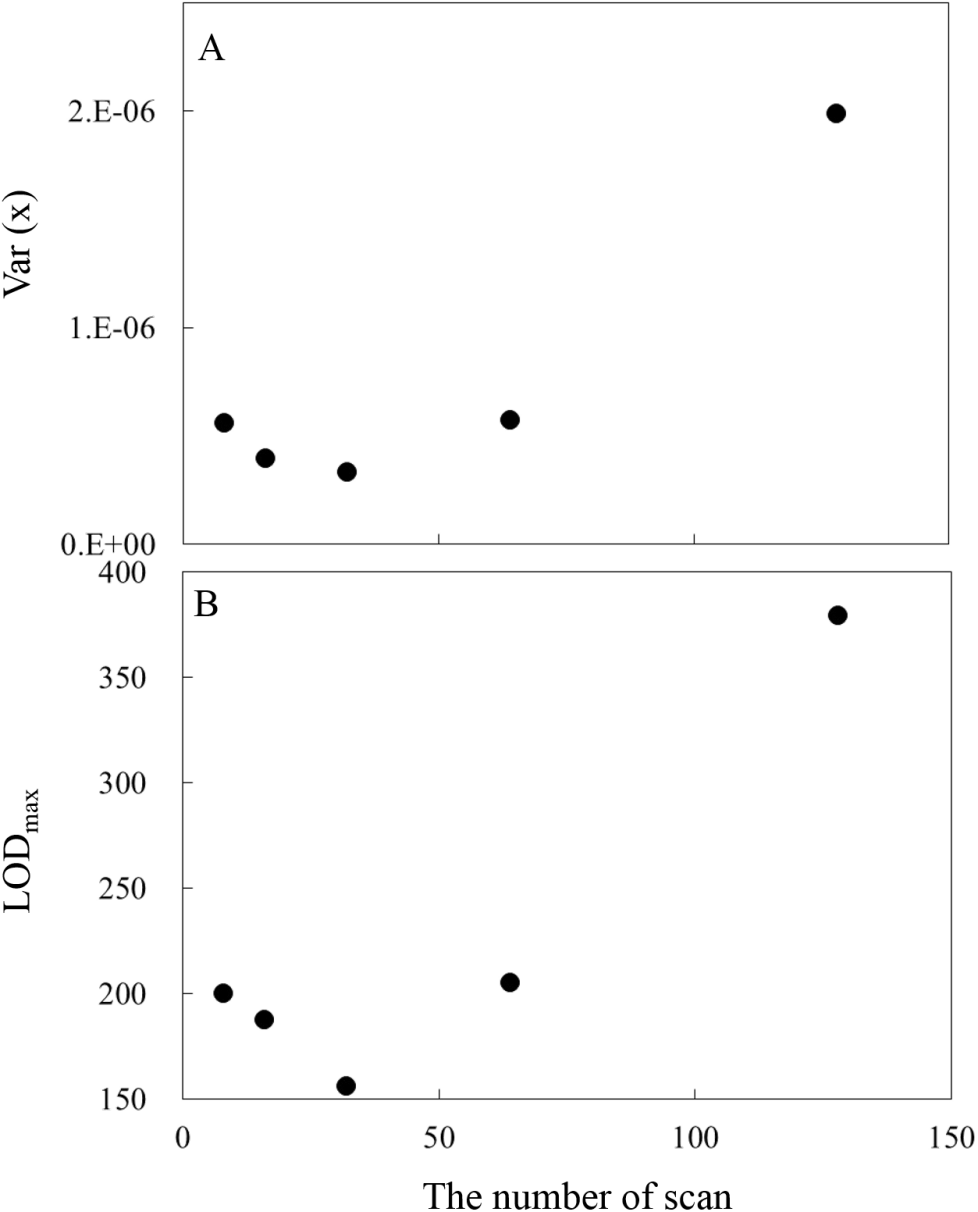
Variations in (A) var(*x*) and (B) LOD_max_ as a function of the number of co-added scan times (spectral pre-treatment: gap-segment second derivative, 23 segments, optical path length: 5 mm, aperture: BRM2065).

The change of var(*x*) as a function of co-added scan time, which directly effect on the S/N ratio, was also investigated. Generally, it is known that the detector noise is inversely proportional to the square root of measurement time. Var(*x*) calculations for 8, 16, 32, 64, 128 scan time (spectral pre-treatment: gap segment second derivative, pathlength: 5 mm, aperture: BR2065) are shown in 9–13 lines from the top in Table 1. Fig 6 shows the change of (A) var(*x*) and (B) LOD_max_ as a function of co-added scan times. In this research, the minimum var(*x*) and LOD_max_ were found when the co-added times was 32 scan, although the co-added times of 64 and 128 scan yield bigger LOD_max_. For higher co-added scan than 32 scan (it took 30s and 60s for measurement for 64 and 128 co-added scan time, respectively), longer periodic noise (i.e. modulation noise such as the variation of incident light intensity due to variations in the light source) have a significant negative effect on the S/N ratio.

### LOD_max_ for FT-NIR spectroscopy

In general, the FT method has the following advantages [21]: (1) the Fellgett advantage resulting from simultaneous measurement of the entire spectral range, which saves time and improves the S/N ratio when spectra are co-added, (2) the Jacquinot advantage, in which greater light throughput is obtained by using a wider diameter aperture and (3) the Cones advantage, associated with a stable reference frequency with very good wavelength accuracy and precision due to the use technique since noise is inversely proportional to the square root of measurement time and has no relation to signal intensity. However, when photon noise (irregular variation of the number of photons reaching the detector) or modulation noise (e.g. variation of incident light intensity due to variations in the light source) are the dominant factors, the multiplexing associated with FT might be a disadvantage. For these reasons, mid-IR spectrometers, in which the detector sensitivity and source energy (when using a glow bar or ceramic) are low because energy throughput is low, generally employ the FT method because the interferometer collects energy over the complete spectral region simultaneously and is therefore more beneficial for a low light throughput system. Conversely, in the UV/VIS region, where detector sensitivity and source energy (such as a deuterium or tungsten lamp) are high, a dispersive grating is used to take advantage of the high energy throughput. Since the NIR region is midway between the UV/VIS and IR regions, both the FT and dispersive grating techniques can be applied. In our case, the best LOD_max_ = 156.2 ppm, in the 60 kinds of spectral measurement condition, was acquired when spectra measured at the 5 mm pathlength using BRM2065 aperture with 32 co-added scan times (second derivative signal (gap-segment 23 smoothing point)). Theoretically, photon noise is proportional to the square root of light intensity from light source. However, in this study, photon noise is not the significant as confirmed by the fact that the var(*x*) decreased with the light intensity as shown in Fig 5. We also showed that the modulation noise was significant when the co-added times was 64 and 128 scans as shown in Fig 6. We should emphasize that the LOD_max_ = 156.2 ppm, acquired in this study for KHP aqueous solution, is not the best result for “FT-NIR spectroscopy”. For example, Ding et al. reported the SEP value of 3.82 ppm for the MIBK ranging between 1–160 ppm using transmission spectra between 5000–4000 cm^-1^ measured by FT-NIR spectrometer with enough *r*^2^ value [7]. The LOD_max_ calculated from the spectra obtained in this study was higher than resulting from such a result due to the external fiber optic cable attached. The work reported herein determined the LOD_max_ associated with the analysis of KHP in water, taking into account some factors which are the especially important for NIR spectroscopy. The spectral measurement condition to provide best LOD_max_ for analyte should be determined taking into account 1. the spectral noise (var(*x*)) and absorptivity (SEN) for the determination of optimal pathlength and 2. detector noise, photon noise and modulation noise. The best condition taking them into account are determined by the measurement changing the pathlength, light intensity from light source and co-added scan times.

## Conclusions

The LOD_max_ for KHP in aqueous solution was determined from the relationship between the sensitivity given in PLS by the inverse of the length of the regression coefficients (SEN), and the variance in instrumental signals (var(*x*)) over the wavenumber range of 6300–5800 cm^-1^ by means of PLS regression analysis. The effects of spectral measurement conditions (pre-treatments, optical path length, light intensity, co-added scan time) on the LOD were all evaluated. It was determined that gap segment second derivative pre-treatment resulted in the best LOD_max_ for all cases and optimal optical path lengths was 4.95 mm. This study found that LOD_max_ of KHP as determined was approximately 150 ppm for our measurement system.
